# Multi-Omics Revealed Key Pathways Related to Soybean (*Glycine max* [L.] Merr.) Seed Hardness

**DOI:** 10.3390/ijms27104473

**Published:** 2026-05-16

**Authors:** Zhen Yuan, Jialiang Liu, Zhilin Zou, Yubo Gao, Zhaoming Qi, Xindong Yao, Dayong Zhang

**Affiliations:** 1 College of Agriculture, Northeast Agricultural University, Harbin 150000, China; 2 Key Laboratory of Soybean Molecular Design Breeding, Northeast Institute of Geography and Agroecology, Chinese Academy of Sciences, Harbin 150081, China

**Keywords:** soybean, seed hardness, GWAS, secondary metabolite biosynthesis

## Abstract

Soybean (*Glycine max* [L.] Merr.) seed hardness is a critical physical trait that dictates processing efficiency and end-product quality, yet the underlying genetic and metabolic regulatory networks remain poorly elucidated. To systematically decipher the mechanisms governing this complex quantitative trait, a multi-omics approach integrating a genome-wide association study (GWAS), transcriptomics, and metabolomics was conducted on a panel of 162 soybean germplasm accessions from Northeast China. Four significant quantitative trait nucleotides (QTNs) on chromosomes 15 and 19 were identified by GWAS. Subsequent RNA-seq and liquid chromatography–mass spectrometry (LC-MS) analyses comparing extreme phenotypes identified 573 differentially expressed genes (DEGs) and 784 differentially accumulated metabolites (DAMs). Joint multi-omics analysis revealed 14 consistently enriched pathways, highlighting the crucial role of secondary metabolite biosynthesis. Notably, *Glyma.19G030500*, which encodes an isoflavone malonyltransferase, was identified as the primary hub gene. These findings offer valuable genomic targets for the marker-assisted breeding of soybean varieties with optimized processing qualities.

## 1. Introduction

Soybean (*Glycine max* [L.] Merr.) is an essential source of vegetable protein and oil worldwide [1]. As the consumption of soy products continues to increase, the nutritional value of foods such as natto, edamame, and tofu is becoming more widely recognized [2]. Consequently, soybean texture and processing suitability are of considerable importance to the market.

Seed hardness is a key physical quality trait of soybean, directly affecting processing efficiency, storage and transportation stability, and the overall quality of end products [3,4,5]. In the processing of traditional soy products such as tofu and soy milk, softer soybeans are ground more thoroughly, which can effectively improve processing efficiency and product yield. In contrast, during natto production, raw soybeans must undergo pre-steaming. Varieties with harder seeds require more stringent steaming parameters, which increases energy consumption and production costs. Furthermore, the intense steaming process tends to cause browning on the product’s surface and often generates a pungent ammonia odor, severely degrading both the appearance and flavor of the natto [3,4,5].

Soybean seed hardness is a complex quantitative trait regulated by the interaction of genetic background and environmental factors, and its phenotypic expression is influenced by a multitude of variables [6]. Previous studies have demonstrated that photo-thermal ecological conditions, precipitation characteristics, seed morphological size, seed coat permeability, imbibition characteristics, seed dormancy, anatomical structure of the seed coat, and the main chemical components of the seed during the entire soybean growth period are all relevant factors determining the seed hardness phenotype [7,8]. Furthermore, research has confirmed a significant correlation between soybean seed hardness and specific agronomic traits. Multiple studies have indicated that seed hardness presents a significant positive correlation with protein content and a negative correlation with oil content [5,9].

Regarding the mapping of genetic loci and the identification of candidate genes regulating soybean seed hardness, previous research has identified multiple quantitative trait loci (QTLs) and genes distributed across various chromosomes. Zhang et al. [4]. utilized recombinant inbred line (RIL) populations derived from a cross between a soft-seeded parent (SS-516) and a hard-seeded parent (Camp) to map loci associated with this trait, successfully identifying two major QTLs designated as Ha1 and Ha2. Subsequent research by the same team identified an additional QTL on chromosome 16 using an independent RIL population [10]. Furthermore, Hirata et al. [11] mapped two QTLs on chromosomes 3 and 6 associated with the hardness of cooked soybeans. Through the fine-mapping of the qHbs3-1 locus, Toda et al. [3] dentified *Glyma03g03360*—a gene highly homologous to plant pectin methylesterase (PME)—as a candidate regulating the hardness of cooked soybean. Additionally, Orazaly et al. [12] identified four molecular markers located on chromosomes 1, 7, 8, and 19 that could be utilized for the marker-assisted selection of soybean seed hardness. Moreover, investigations utilizing a Chinese mini-core germplasm collection [4] highlighted two QTLs on chromosome 15 as critical genomic regions harboring hub genes that regulate soybean seed hardness.

In addition, relevant studies have elucidated the regulatory mechanisms of genes involved in cell wall lignin biosynthesis, establishing that *GmCAD4* is a key rate-limiting enzyme gene for lignin synthesis in soybean; its participation in the regulation of seed hardness has been widely verified [13]. Furthermore, research by Ren et al. [14] demonstrated that *Glyma.02G307000* encodes a pectate lyase-like superfamily protein, which is hypothesized to influence the seed hardness phenotype by modulating pectate lyase activity.

Although numerous genes have been demonstrated to be associated with soybean seed hardness, few studies have attempted to elucidate the phenotypic expression of this trait through key regulatory or metabolic pathways. In this study, a set of 162 soybean germplasm accessions from Northeast China was utilized. Genome-wide association analysis, and transcriptome and metabolome multi-omics techniques were integrated to evaluate soybean seed hardness and investigate the underlying genetic mechanisms. The objective of this study is to explore the genetic loci, key regulatory genes, and differential metabolites significantly associated with soybean seed hardness, and to systematically analyze the regulatory networks controlling seed hardness formation.

## 2. Results

### 2.1. GWAS Analysis for Soybean Seed Hardness

**Figure 1:**
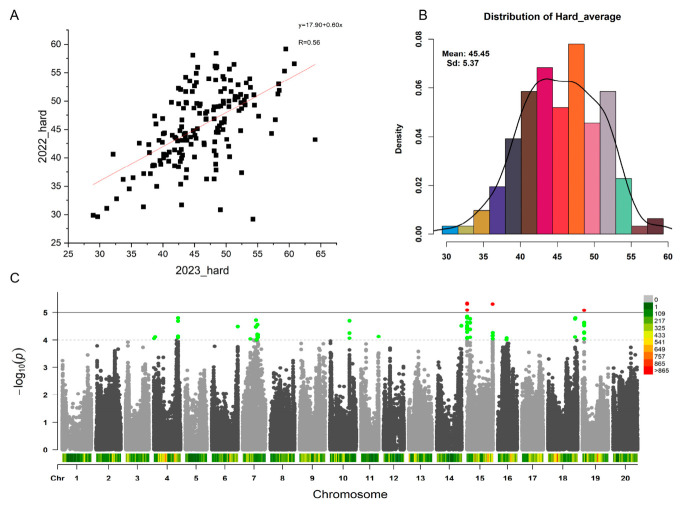
GWAS analysis for soybean seed hardness. (**A**) Correlation analysis for soybean seed hardness of 162 varieties between 2022 and 2023. (**B**) Distribution of the average seed hardness value used fow GWAS of 2022 and 2023. (**C**) Manhattan plot of seed hardness, green points are the potential QTNs, red points are the significant QTNs. Colored belt indicates the SNP density of each chromesome.

In this study, a panel of 162 soybean varieties was utilized to conduct a genome-wide association study (GWAS) on seed hardness employing the FarmCPU model. Phenotypic evaluations across two environments revealed a mean seed hardness (SH) of 45.54 in 2022 (ranging from 29.21 to 59.20) and 45.94 in 2023 (ranging from 28.94 to 64.10). The coefficient of variation (CV) for this trait was 14.5% and 13.5% in 2022 and 2023, respectively (Table 1). A significant positive correlation was observed between the phenotypic data of the two years (r = 0.56; Figure 1A), indicating that seed hardness is substantially governed by genetic factors. For the association analysis, mean SH values across both environments were calculated; these values exhibited a continuous, normal distribution (Figure 1B), successfully satisfying the prerequisite statistical assumptions for GWAS. Furthermore, principal component analysis (PCA) demonstrated extensive genetic diversity and distance within the assembled germplasm (Supplementary Figure S1), thereby confirming the robustness and reliability of utilizing this 162-accession panel for association mapping.

Four significant quantitative trait nucleotides (QTNs) were identified by the FarmCPU model at a significance threshold of 5. Three of these QTNs mapped to chromosome 15, while the remaining QTN was located on chromosome 19 (Figure 1C). To mitigate the risk of false-negative associations, the threshold was subsequently relaxed to four to encompass all putative QTNs. A 200 kb flanking region surrounding each QTN was defined as the target interval for gene identification. Consequently, a total of 256 genes distributed across chromosomes 4, 7, 10, 11, 15, 16, 18, and 19 were designated as candidate genes regulating soybean seed hardness (Supplementary Table S1).

### 2.2. RNA-Seq Analysis of Seed Hardness

**Figure 2:**
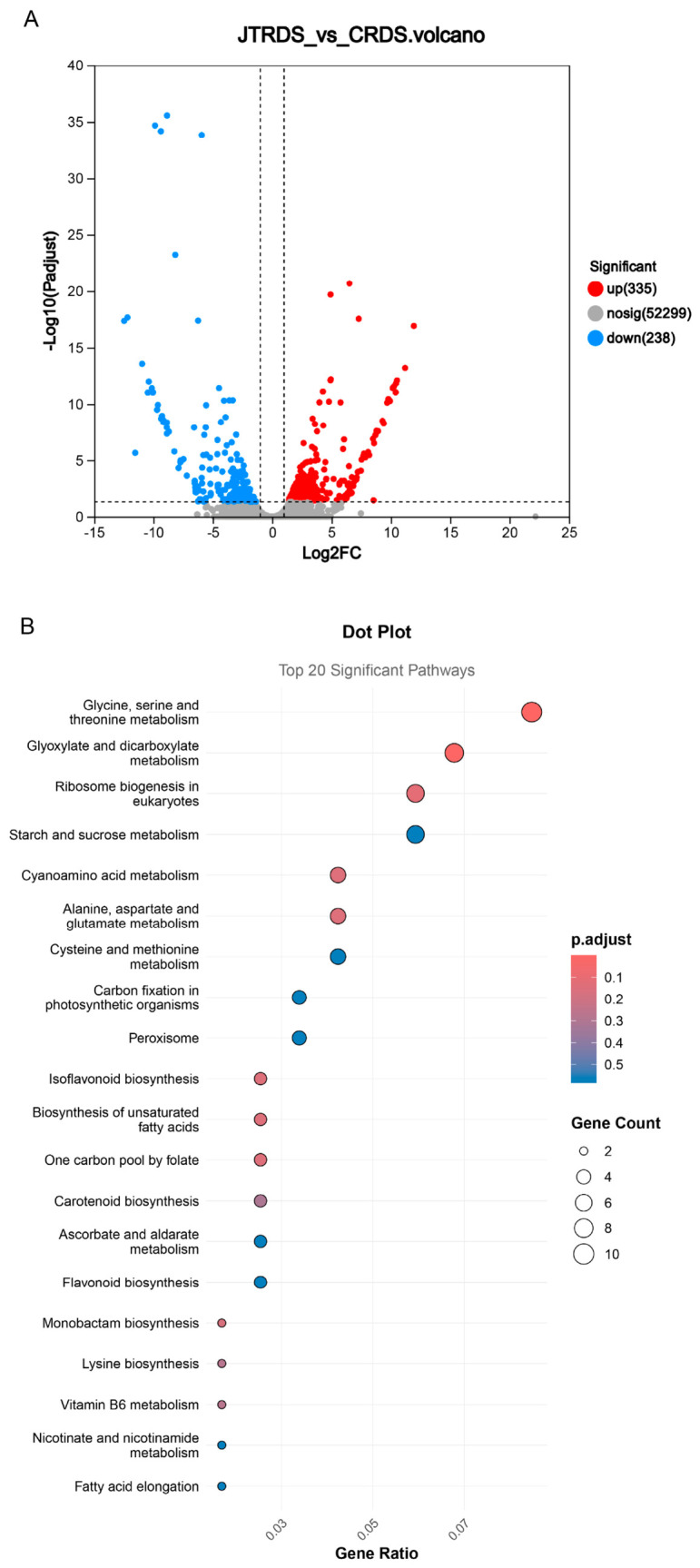
RNA analysis for JT vs. C varieties. (**A**) Volcano plot of JT vs. C varieties, red points are the up-regulated genes and the blue points are the down-regulated genes. (**B**) Top 20 significant pathways by KEGG enrichment of the DEGs.

To identify hub genes associated with seed hardness, two contrasting varieties were selected for RNA sequencing (RNA-seq) based on their phenotypic stability across both years: JT, exhibiting a high mean seed hardness of 59.32, and C, exhibiting a low mean seed hardness of 29.42. Three biological replicates of each variety were sequenced using the Illumina platform, yielding 6 to 7 Gb of clean bases per sample. Following data filtration (adjusted *p* < 0.001 and |log2FC| ≥ 1), a total of 573 differentially expressed genes (DEGs) were identified between the two varieties (Supplementary Table S2). Relative to variety C, 335 genes were up-regulated and 238 genes were down-regulated (Figure 2A). Kyoto Encyclopedia of Genes and Genomes (KEGG) pathway enrichment analysis categorized these DEGs into various metabolic networks. The top 10 significantly enriched pathways included glycine, serine, and threonine metabolism; glyoxylate and dicarboxylate metabolism; ribosome biogenesis in eukaryotes; cyanoamino acid metabolism; alanine, aspartate, and glutamate metabolism; isoflavonoid biosynthesis; biosynthesis of unsaturated fatty acids; one carbon pool by folate; monobactam biosynthesis; and lysine biosynthesis (Figure 2B).

### 2.3. Changes in Metabolic Levels

**Figure 3:**
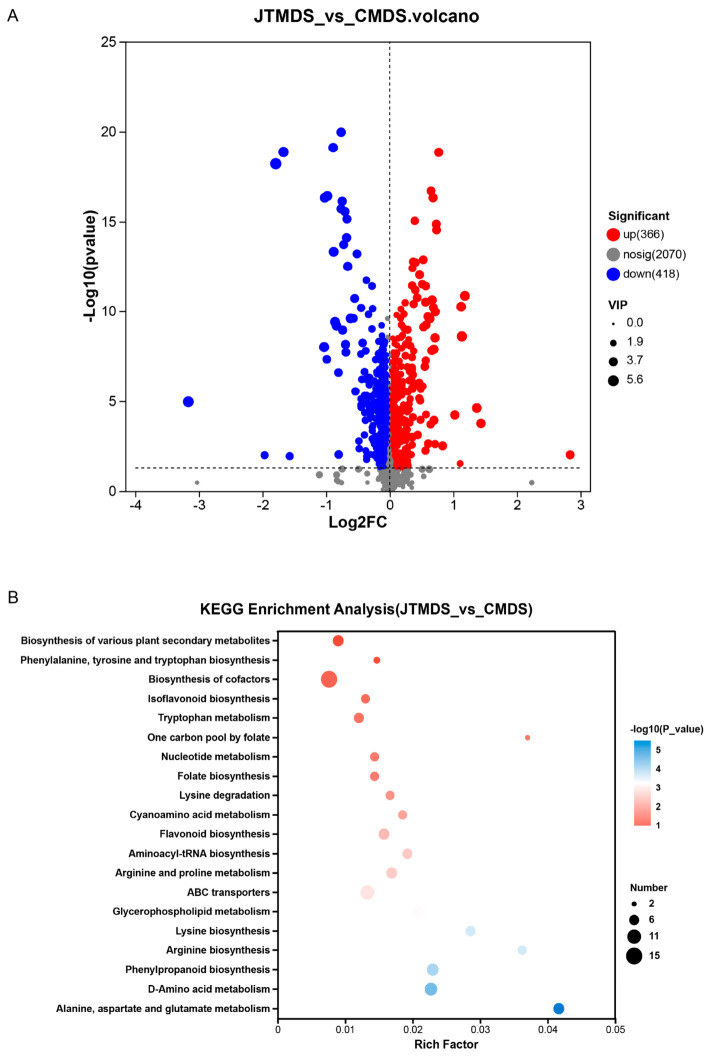
LC-MS analysis for JT and C varieties. (**A**) Volcano plot of DAMs, red points are the up-regulated metabolites and the blue points are the down-regulated metabolites. (**B**) Top 20 pathways enriched by KEGG analysis between JT and C DAMs.

Dry seeds of varieties JT and C were utilized for LC-MS analysis. A total of 2854 distinct metabolites were detected across both positive and negative ion modes. Following data filtration (using thresholds of *p* < 0.05 and VIP > 1), 366 significantly up-regulated and 418 significantly down-regulated differentially accumulated metabolites (DAMs) were identified (Figure 3A). Kyoto Encyclopedia of Genes and Genomes (KEGG) pathway enrichment analysis of these significant DAMs revealed that the top 10 enriched pathways included: alanine, aspartate, and glutamate metabolism; D-amino acid metabolism; phenylpropanoid biosynthesis; lysine biosynthesis; arginine biosynthesis; glycerophospholipid metabolism; ABC transporters; aminoacyl-tRNA biosynthesis; arginine and proline metabolism; and flavonoid biosynthesis (Figure 3B).

### 2.4. Joint Analysis for GWAS, Transcriptome and Metabolomics

**Figure 4:**
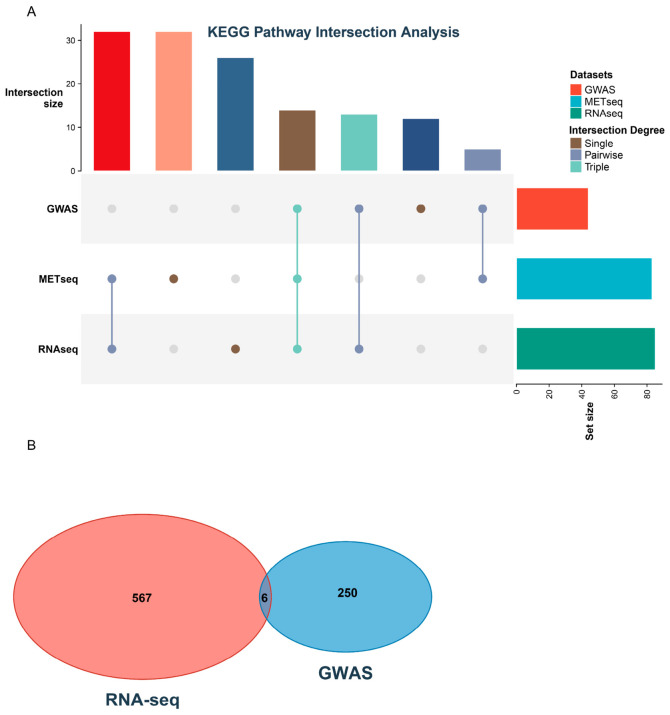
Joint analysis for GWAS, transcriptome and metabolomics. (**A**) Up-set plot for the pathways enriched based on GWAS candidate genes, DEGs and DAMs. (**B**) Venn plot of GWAS candidate genes and DEGs.

Independent Kyoto Encyclopedia of Genes and Genomes (KEGG) pathway analyses were conducted for the GWAS candidate genes, significant DEGs, and significant DAMs. The GWAS candidate genes were categorized into 44 pathways, whereas the DEGs and DAMs mapped to 85 and 83 pathways, respectively. Among these, 14 pathways were consistently annotated across all three datasets (Figure 4A). With the exception of one pathway assigned to the Environmental Information Processing category, all other shared pathways were classified under Metabolism. An overlap of six genes was identified between the GWAS candidate genes and the significant DEGs (Figure 4B, Supplementary Table S2). Within this subset, *Glyma.19G030500* was annotated to pathways map00943 and map00944, and *Glyma.11G254300* was annotated to map00920, which is associated with energy metabolism. Consequently, *Glyma.19G030500* is proposed as a crucial hub gene regulating soybean seed hardness, with pathways map00943 and map00944 playing significant roles in the phenotypic formation of this trait.

### 2.5. qPCR for Candidate Gene and Haplotype Analysis

**Figure 5:**
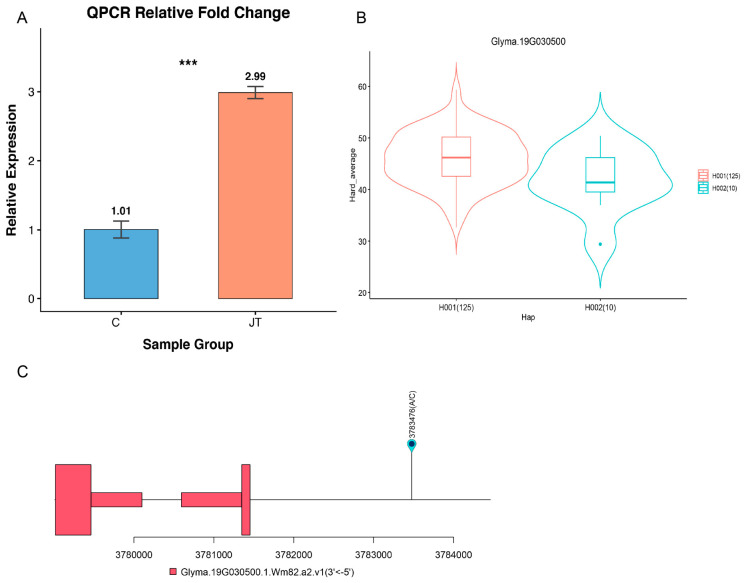
qPCR and haplotype analysis for gene *Glyma.19G030500*. (**A**) Relative gene expression of gene *Glyma.19G030500* in JT and C. *** means *p* < 0.001 (**B**) Soybean seed hardness data within different haplotypes. Numbers meaning the varieties number carrying the specific haplotype. (**C**) Haplotype model of the variant.

To verify the candidate gene expression in the two varieties, qPCR was used for analysis. The relative gene expression of candidate gene *Glyma.19G030500* was about three times bigger (Figure 5A) in high-seed-hardness variety C than the low-seed-hardness variety JT, whereas the foldchange according to the RNA seq data was 4.3.

Haplotype analysis based on the candidate gene indicates that the variant loci was located on the promoter area of the gene (Figure 5C). When we removed the heterozygous and the missing value, two haplotypes were identified in the nature population panel: H001 (the varieties carrying base A) and H002 (varieties carrying base C) (Figure 5B). There were 125 varieties grouped into H001 with the average soybean seed hardness of 46.0, whereas there were ten varieties grouped into H002 with the average soybean seed hardness of 41.8.

## 3. Discussion

### 3.1. The Seed Hardness and Hard Seededness

In the present study, a clear distinction is established between soybean seed hardness and hard-seededness. Hard-seededness is an adaptive trait that can extend seed longevity, but it is frequently detrimental to seed germination and seedling emergence [15,16]. In contrast, the seed hardness evaluated herein represents a physical textural trait. Hard-seededness is primarily an adaptive, ecological survival trait prevalent in wild soybean (*Glycine soja*) and numerous other wild leguminous species. It functions as a mechanism of physical dormancy, isolating the embryo from the surrounding environment by rendering the seed coat completely impermeable to water and gas exchange. This impermeability ensures long-term seed viability and prevents premature germination under transient or suboptimal environmental conditions, effectively extending seed longevity in the soil bank. At the anatomical and molecular levels, hard-seededness is exclusively dictated by the highly specialized structural characteristics of the seed coat (testa) and its outermost protective layers. The impermeability barrier is established by the dense compaction of the epidermal macrosclereid (palisade) cell layer and the underlying hypodermal osteosclereid (hourglass) cells. The extracellular matrix of this region is heavily fortified with cuticular substances, including suberin, cutin, and complex waxes, which create a highly hydrophobic shield. Whereas hard-seededness is predominantly governed by seed coat characteristics, soybean seed hardness is largely influenced by the internal chemical composition of the seed. The “seed hardness” evaluated for processing suitability is a physical, textural trait that is largely independent of the initial permeability of the testa. Seed hardness represents the internal resistance of the cotyledonary tissue to mechanical deformation, compression, or puncture. In contemporary evaluations, this trait is quantified using precision texture analyzers (e.g., TA.XTplusC) that utilize a puncture probe to measure the mechanical work (measured in g·mm or Newtons) required to penetrate the mature, dry, or hydrated seed to a specific depth. Crucially, while hard-seededness profoundly restricts water entry and inhibits germination, elevated seed hardness does not inherently compromise seed imbibition kinetics, nor does it negatively impact overall seed vigor or seedling emergence rates. Cultivated soybean panels exhibiting vast phenotypic variation in seed hardness (with coefficients of variation exceeding 13–14%) frequently demonstrate highly stable, uniform seedling emergence across diverse environments, confirming that internal cotyledonary hardness is physiologically uncoupled from the physical dormancy imposed by a waterproof seed coat. This study utilized an assembled panel of 162 soybean varieties widely cultivated in Northeast China. Although substantial phenotypic variation in seed hardness was observed among the accessions—with coefficient of variation (CV) values of 14.5% in 2022 and 13.5% in 2023—seedling emergence rates remained highly stable across both environments. Therefore, these observations indicate that soybean seed hardness is physiologically distinct from hard-seededness, and that elevated seed hardness does not negatively impact seed vigor.

### 3.2. Multi-Omics Revealed the Key Pathway and Hub Gene for Regulating Soybean Seed Hardness

Although omics technologies have made significant advancements over the last decade, limitations remain in utilizing single-omics approaches to explain specific phenotypes [17]. In this study, a panel of 162 varieties from Northeast China was assembled; principal component analysis (PCA) indicated no apparent clustering among the accessions. Furthermore, the varieties exhibiting the highest and lowest mean seed hardness were selected for transcriptomic and metabolomic analyses. Because these two varieties were selected from a natural population, they differ in numerous agronomic traits beyond seed hardness. Consequently, the most substantial fold-changes in DEGs and DAMs may be related to these varying traits rather than seed hardness alone. To mitigate the confounding effects arising from the genetic backgrounds of the selected accessions, this study integrates GWAS, transcriptomics, and metabolomics to reliably elucidate the key regulatory pathways governing seed hardness.

The most significantly enriched pathways based on the DAMs were classified into lipid metabolism, global and overview maps, membrane transport, signal transduction, amino acid metabolism, and the biosynthesis of other secondary metabolites. This suggests that seed hardness may be influenced by oil content, protein content, and secondary metabolite accumulation. Previous studies have similarly demonstrated a correlation between soybean seed hardness and internal nutritional components [18,19]. Thus, the metabolomic analysis results are considered highly reliable.

Consistent with previous findings, pathways related to amino acid and starch metabolism—such as “alanine, aspartate, and glutamate metabolism” and “starch and sucrose metabolism”—were significantly enriched in the DEG analysis [9]. However, given the substantial differences in the genetic backgrounds of the two samples selected for the transcriptomic analysis, it is challenging to definitively identify the specific pathways influencing seed hardness using RNA-seq data alone.

GWAS results indicate that there were a few QTLs distributed on chromosomes 4, 6, 7, 10, 11, 15, 16, 18, and 19. Previously, Zhang et al. [5] identified a significant SNP, Q-15-0087770, associated with seed hardness via GWAS in a Chinese mini core collection using 1536 SNPs. Additionally, two stable QTLs related to seed hardness were mapped to linkage groups L (Chr 19) and D1a (Chr 1) by Zhang et al. [4]. Although the present GWAS identified QTLs on chromosomes 15 and 19, their genomic positions differ from those previously reported. These findings indicate that soybean seed hardness is a complex, polygenic trait regulated by diverse genetic loci.

Upon integrating the pathways identified independently by the three methods in this study, 14 pathways were consistently enriched across all three datasets. These pathways are primarily categorized into amino acid metabolism, the biosynthesis of other secondary metabolites, lipid metabolism, and the metabolism of terpenoids and polyketides, among others. Further integration of the GWAS candidate genes and RNA-seq DEGs identified an overlap of six genes. Notably, *Glyma.19G030500*, which is annotated to map00943 (isoflavonoid biosynthesis) and map00944 (flavone and flavonol biosynthesis), is proposed as the primary hub gene regulating soybean seed hardness.

### 3.3. Potential Mechanism of Two Pathways Regulating Soybean Seed Hardness

*Glyma.19G030500*, the hub gene we identified, encodes an enzyme flavonol 7-O-beta-D-glucoside malonyltransferase according to phytozome [20]. Our integrated transcriptomic and metabolomic analysis reveals a significant negative correlation between the expression of isoflavone 7-O-glucoside-6″-O-malonyltransferase (EC 2.3.1.115) and soybean seed hardness. Notably, softer seeds exhibited a marked accumulation of naringenin, a pivotal intermediate at the junction of the isoflavonoid (map00943) and flavone/flavonol (map00944) biosynthesis pathways. Previous studies showed that lignin and phenylpropanoid metabolism pathways affected the components of the cell wall and led to hard seededness [21,22,23,24,25].

Although there is a difference between hard seededness and seed hardness, we hypothesize that the upregulation of EC 2.3.1.115 creates a robust metabolic sink at the terminus of map00943, driving upstream carbon flux toward isoflavonoid production. The concurrent accumulation of naringenin indicates a metabolic bottleneck, suggesting that carbon flux is aggressively diverted away from downstream structural branches, particularly the synthesis of proanthocyanidins via map00944 and lignin via the general phenylpropanoid pathway. The consequent reduction in these rigid, cross-linked cell wall polymers—coupled with the altered intracellular osmotic potential caused by the accumulation of highly soluble malonylated isoflavonoids—provides a comprehensive biochemical rationale for the observed softening of the seed phenotype.

While transcriptomic shifts and enzyme kinetics delineate the potential trajectories of carbon flux, the actual execution of these metabolic reallocations—particularly the physical assembly of the cell wall—is intricately modulated by intracellular redox signaling networks. The integration of reactive oxygen species (ROS) and reactive nitrogen species (RNS) dynamics is essential for bridging the conceptual gap between genetic potential and the ultimate biophysical phenotype. Consequently, incorporating the redox paradigm proposed by Ali et al. [26] enriches the mechanistic interpretation of seed hardness. This framework suggests that the final physical texture of the soybean is dictated not solely by the availability of storage compounds and competitive transcriptomic flux, but also by the precise spatiotemporal deployment of ROS and RNS required to forge structural cross-links within the cell wall [26].

Applying this advanced spatial perspective to the soybean cotyledon resolves critical ambiguities regarding the localized regulation of metabolic flux and seed hardness [27]. Traditional bulk tissue analyses operate on the assumption that biochemical competition—such as that between lignin and isoflavonoid biosynthesis—occurs uniformly across all cotyledonary cells. However, recent spatial transcriptomic mapping of mid-maturity wild soybean (*Glycine soja*) seeds decisively refutes this assumption, revealing a profound structural and metabolic dichotomy within the embryonic parenchyma. Specifically, the adaxial parenchyma cells, oriented toward the seed interior, exhibit substantial enrichment in lipid metabolism pathways, functioning as localized hubs for oil body accumulation. In stark contrast, the abaxial parenchyma cells, oriented toward the exterior, are almost exclusively specialized for protein metabolism, amino acid biosynthesis, and the assembly of dense protein storage vacuoles [28]. This cell-type-specific compartmentalization provides indispensable mechanistic depth, demonstrating that soybean seed hardness is not merely a bulk biochemical average, but rather the culmination of an exquisite, three-dimensional spatial and metabolic architecture.

## 4. Materials and Methods

### 4.1. Experimental Site and Plant Materials

The plant materials utilized in this study comprised a panel of 162 soybean germplasm accessions collected from Northeast China, provided by the Soybean Research Group of Northeast Agricultural University. Field experiments were conducted at the Xiangyang Farm experimental station (126°54′36.778″ E, 45°46′17.96″ N) during the 2022 and 2023 growing seasons. The experimental plots consisted of 60 rows, featuring a row length of 3 m, a row spacing of 65 cm, and an intra-row plant spacing of 6 cm. The experimental site is characterized by a mid-temperate semi-humid continental monsoon climate, with typical black soil predominating. The baseline nutrient profile of the plow layer (0–20 cm depth) was determined as follows: pH 6.96, alkali-hydrolyzable nitrogen 214.67 mg kg^−1^, organic matter 14.74 g kg^−1^, available phosphorus 41.76 mg kg^−1^, and available potassium 180.33 mg kg^−1^. All additional agronomic management and field practices were carried out in accordance with standard local commercial soybean cultivation protocols.

### 4.2. Soybean Seed Hardness Measurement

A TA.XTplusC texture analyzer (Stable Micro Systems, Godalming, UK) equipped with a 2 mm diameter probe was utilized to determine the seed hardness of the 162 soybean varieties at the mature stage using the puncture method [4,9]. The instrument parameters were configured as follows: a puncture speed of 1 mm/s, a penetration depth of 3 mm, and a trigger force of 0.5 g to initiate data recording via the Texture Exponent 32 software (version 6.2) upon probe contact with the sample surface. A force (g) versus time (s) curve was generated for each puncture event. Following each test, the probe automatically retracted to its initial position to allow for sample replacement. For each variety, 20 individual seeds were measured as replicates. The mechanical work, W (g·mm), required for the probe to penetrate the seed to a depth of 2 mm was calculated and utilized as the primary parameter to evaluate seed hardness. A custom macro within the Texture Exponent 32 software was programmed to automate data extraction from the generated measurement curves.

### 4.3. Genome-Wide Association Study (GWAS) Analysis

Single nucleotide polymorphism (SNP) genotyping data for the 162 soybean varieties were obtained from Northeast Agricultural University, as described by Luan et al. [29]. Following quality control filtering (minor allele frequency [MAF] ≥ 0.05, missing data rate ≤ 10, and linkage disequilibrium [LD] pruning), a total of 200,835 high-quality SNPs were retained for the association analysis. The genome-wide association study (GWAS) was subsequently conducted using the FarmCPU model implemented in the R package Rmvp (1.4.6) [30].

### 4.4. RNA-Seq Analysis

Dry seeds of varieties C and JT were ground into a fine powder in liquid nitrogen, with three biological replicates processed per variety. Total RNA was extracted from the homogenized tissue using TRIzol^®^ Reagent, with the procedure performed by Shanghai Majorbio Bio-pharm Technology Co., Ltd. (Shanghai, China) in strict accordance with the manufacturer’s protocols. RNA integrity and quality were subsequently evaluated using an Agilent 5300 Bioanalyzer (Agilent Technologies, Santa Clara, CA, USA), and quantification was conducted with a NanoDrop ND-2000 spectrophotometer (NanoDrop Technologies, Wilmington, DE, USA). Only high-quality RNA samples—defined by an OD260/280 = 1.8–2.2, OD260/230 ≥ 2.0, RQN ≥ 6.5, 28S:18S ≥ 1.0, >1 μg—were selected for the construction of the sequencing libraries. Subsequent transcriptomic data analysis was executed utilizing the Majorbio Cloud platform (www.majorbio.com) [31]. To identify DEGs (differential expression genes) between two different samples, the expression level of each transcript was calculated according to the transcripts per million reads (TPM) method. RSEM was used to quantify gene abundances. Essentially, differential expression analysis was performed using the DESeq2 or DEGseq. DEGs with |log2FC| ≥ 1 and FDR < 0.05(DESeq2) or FDR < 0.001(DEGseq) were considered to be significantly different expressed genes. In addition, KEGG was performed to identify which DEGs were significantly enriched in metabolic pathways at Bonferroni-corrected *p*-value < 0.05 compared with the whole-transcriptome background. KEGG pathway analysis was carried out by Python scipy software (1.0.0).

### 4.5. Metabolome Analysis

A 100 mg aliquot of dry soybean seed (with six biological replicates processed per variety) was transferred into a 2 mL centrifuge tube containing a 6 mm diameter grinding bead. Metabolite extraction was initiated by adding 800 μL of an extraction solvent mixture (methanol:water, 4:1, *v*/*v*) spiked with four internal standards (including 0.02 mg/mL L-2-chlorophenylalanine). The samples were homogenized using a Wonbio-96c frozen tissue grinder (Shanghai Wanbo Biotechnology Co., Ltd., Shanghai, China) for 6 min at −10 °C and 50 Hz. This was immediately followed by low-temperature sonication for 30 min at 5 °C and 40 kHz. Subsequently, the homogenates were incubated at −20 °C for 30 min and then centrifuged at 13,000× *g* for 15 min at 4 °C. The resulting supernatant was carefully transferred to an autosampler vial for liquid chromatography–tandem mass spectrometry (LC-MS/MS) analysis. Downstream metabolomic data analysis was executed utilizing the Majorbio Cloud platform (www.majorbio.com). The pretreatment of LC/MS raw data was performed by Progenesis QI (Version 2.1) (Waters Corporation, Milford, MA, USA) software, and a three-dimensional data matrix in CSV format was exported. The information in this three-dimensional matrix included: sample information, metabolite name and mass spectral response intensity. Internal standard peaks, as well as any known false positive peaks (including noise, column bleed, and derivatized reagent peaks), were removed from the data matrix, deredundant and peak pooled. At the same time, the metabolites were identified by searching the database, and the main databases were the HMDB (http://www.hmdb.ca/ accessed on 9 May 2023), Metlin (https://metlin.scripps.edu/ accessed on 9 May 2023) and the self-compiled Majorbio Database (MJDB) of Majorbio Biotechnology Co., Ltd. (Shanghai, China). The data matrix obtained by searching the database was uploaded to the Majorbio cloud platform (https://cloud.majorbio.com accessed on 9 May 2023) for data analysis. Fistly, the data matrix was pre-processed, as follows: At least 80% of the metabolic features detected in any set of samples were retained. After filtering, the minimum value in the data matrix was selected to fill the missing value and each metabolic signature was normalized to the sum. To reduce the errors caused by sample preparation and instrument instability, the response intensities of the sample mass spectrometry peaks were normalized using the sum normalization method, to obtain the normalized data matrix. Meanwhile, the variables of QC samples with relative standard deviation (RSD) > 30% were excluded and log10 logarithmicized, to obtain the final data matrix for subsequent analysis. Perform variance analysis on the matrix file after data preprocessing. The R package “ropls” (Version 1.6.2) was used to perform principal component analysis (PCA) and orthogonal least partial squares discriminant analysis (OPLS-DA), and seven-cycle interactive validation evaluating the stability of the model. The metabolites with VIP > 1, *p* < 0.05 were determined as significantly different metabolites based on the Variable importance in the projeciton (VIP) obtained by the OPLS-DA model and the *p*-value generated by Student’s *t* test. Differential metabolites among two groups were mapped into their biochemical pathways through metabolic enrichment and pathway analysis based on the KEGG database (http://www.genome.jp/kegg/ accessed on 9 May 2023). These metabolites could be classified according to the pathways they involved or the functions they performed. Enrichment analysis was used to analyze whether a group of metabolites appears in a function node or not. The principle was that the annotation analysis of a single metabolite develops into an annotation analysis of a group of metabolites. Python packages “scipy.stats” (https://docs.scipy.org/doc/scipy/ accessed on 9 May 2023) was used to perform enrichment analysis to obtain the most relevant biological pathways for experimental treatments.

### 4.6. Statistical Analysis of Data

Upset diagram and Venn diagram were analyzed and ploted in R software (4.5.1) (R Core Team (2025)) with “ComplexUpset” and “VennDiagram” respectively. Haplotype analysis used the R package “geneHapR” and all the codes followed the package guideline.

## 5. Conclusions

This study integrated genomic, transcriptomic, and metabolomic analyses to elucidate the complex regulatory networks governing soybean seed hardness. Through GWAS, four significant QTNs associated with this trait were mapped to chromosomes 15 and 19. The subsequent integration of multi-omics data identified *Glyma.19G030500* as a critical hub gene operating at the biochemical junction of the isoflavonoid and flavone/flavonol biosynthesis pathways. Ultimately, these findings provide a robust theoretical foundation and specific genetic loci for the marker-assisted selection and genetic improvement of soybean varieties tailored for specialized food processing.

## Figures and Tables

**Table 1:** Descriptive statistics of seed hardness in 2022 and 2023.

Year	N	Mean	SD	SE	Min	Max	CV
2022	162	45.51	6.62	0.52	29.21	59.2	14.5
2023	162	45.94	6.2	0.49	28.94	64.1	13.5

## Data Availability

All the original data supporting this study are available on request from the corresponding author, X.Y., upon reasonable request. The RNA sequencing data can be accessed via the link: https://ngdc.cncb.ac.cn/gsa/browse/CRA042157 (accessed 29 April 2026).

## Supplementary Materials

The following supporting information can be downloaded at: https://www.mdpi.com/article/10.3390/ijms27104473/s1.
