# Supplementary material for: Multi-Omics Revealed Key Pathways Related to Soybean (Glycine max [L.] Merr.) Seed Hardness
Source: Int J Mol Sci. 2026 May 16;27(10):4473. doi: 10.3390/ijms27104473 (PMC13207912; doi:10.3390/ijms27104473)
Supplement: Supplementary file 1 [file ijms-27-04473-s001.zip › ijms-4266354-supplementary.pdf]

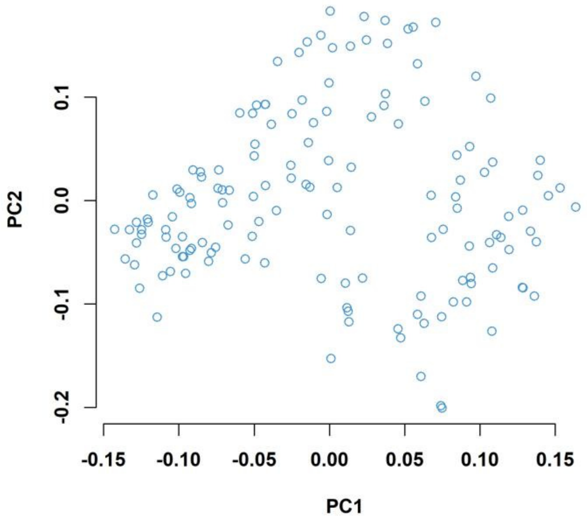

Suppl. Figure S1 Principal component analysis for 162 varieties used in this study

Suppl. Table S1 Candidate genes among the QTL area

Chr	Start	End	LOCUS TAG
Chr04	308124	309067	Glyma.04G003700
Chr04	314908	318098	Glyma.04G003800
Chr04	318939	321963	Glyma.04G003900
Chr04	348291	349284	Glyma.04G004100
Chr04	361722	364849	Glyma.04G004200
Chr04	381212	382422	Glyma.04G004400
Chr04	384957	387999	Glyma.04G004500
Chr04	294705	296341	Glyma.04G003600
Chr04	324705	335134	Glyma.04G004000
Chr04	373953	378634	Glyma.04G004300
Chr04	392963	396685	Glyma.04G004600
Chr04	2674641	2681120	Glyma.04G033600
Chr04	2687701	2689839	Glyma.04G033800
Chr04	2704888	2708138	Glyma.04G033900
Chr04	2714105	2719171	Glyma.04G034000
Chr04	2720426	2721270	Glyma.04G034100
Chr04	2744309	2745136	Glyma.04G034300
Chr04	2751147	2752368	Glyma.04G034400
Chr04	2753362	2755246	Glyma.04G034500
Chr04	2762601	2763662	Glyma.04G034600
Chr04	2770694	2784094	Glyma.04G034700
Chr04	2680425	2687255	Glyma.04G033700
Chr04	2734770	2742608	Glyma.04G034200
Chr04	47289746	47297214	Glyma.04G200400
Chr04	47311582	47315663	Glyma.04G200500
Chr04	47352796	47360810	Glyma.04G200800
Chr04	47397562	47397949	Glyma.04G201100
Chr04	47403086	47411795	Glyma.04G201200
Chr04	47421729	47428563	Glyma.04G201300
Chr04	47280047	47286895	Glyma.04G200300
Chr04	47349720	47352523	Glyma.04G200700
Chr04	47363745	47372595	Glyma.04G200900
Chr04	47375498	47382776	Glyma.04G201000
Chr04	47316151	47321158	Glyma.04G200600
Chr06	50957993	50969115	Glyma.06G320900
Chr06	50974137	50985004	Glyma.06G321000
Chr06	50984727	50984957	Glyma.06G321100
Chr06	50989288	50993485	Glyma.06G321200
Chr06	50996993	50999646	Glyma.06G321300
Chr06	51007221	51008804	Glyma.06G321500
Chr06	51023725	51025677	Glyma.06G321600
Chr06	51030030	51034911	Glyma.06G321700
Chr06	51037094	51039423	Glyma.06G321800
Chr06	51040796	51041580	Glyma.06G321900
Chr06	51042976	51045446	Glyma.06G322000
Chr06	51046118	51047447	Glyma.06G322100
Chr06	51058001	51065788	Glyma.06G322300
Chr06	51063411	51063768	Glyma.06G322400
Chr06	51001786	51006353	Glyma.06G321400
Chr06	51048637	51055248	Glyma.06G322200
Chr07	15122531	15127433	Glyma.07G126600
Chr07	15150229	15150444	Glyma.07G126700
Chr07	15101055	15107128	Glyma.07G126500
Chr07	25694302	25696008	Glyma.07G166100
Chr07	25654885	25687841	Glyma.07G166000
Chr07	25704908	25707949	Glyma.07G166200
Chr07	26166480	26166823	Glyma.07G166600

Chr	Start	End	LOCUS TAG
Chr07	26923234	26924152	Glyma.07G167000
Chr07	26975615	26976121	Glyma.07G167100
Chr07	28509830	28511830	Glyma.07G169100
Chr07	28512749	28514533	Glyma.07G169200
Chr07	28514698	28515694	Glyma.07G169300
Chr07	28527364	28528795	Glyma.07G169400
Chr07	28599162	28603639	Glyma.07G169500
Chr07	28603984	28605373	Glyma.07G169600
Chr07	28607205	28619156	Glyma.07G169700
Chr07	28620736	28621520	Glyma.07G169800
Chr07	28623911	28624823	Glyma.07G169900
Chr07	29904143	29905331	Glyma.07G172100
Chr07	29897101	29901738	Glyma.07G172000
Chr07	29986095	29987051	Glyma.07G172300
Chr07	30030699	30035054	Glyma.07G172400
Chr10	38372070	38378539	Glyma.10G148300
Chr10	38379206	38380060	Glyma.10G148400
Chr10	38381421	38382250	Glyma.10G148500
Chr10	38415381	38418722	Glyma.10G148700
Chr10	38427372	38430979	Glyma.10G148900
Chr10	38431647	38436740	Glyma.10G149000
Chr10	38441580	38441963	Glyma.10G149100
Chr10	38464899	38467826	Glyma.10G149200
Chr10	38468901	38469373	Glyma.10G149300
Chr10	38469764	38473037	Glyma.10G149400
Chr10	38493040	38499888	Glyma.10G149500
Chr10	38382588	38389222	Glyma.10G148600
Chr10	38420217	38425350	Glyma.10G148800
Chr10	38559852	38564099	Glyma.10G150200
Chr10	38573748	38575248	Glyma.10G150400
Chr10	38586097	38588519	Glyma.10G150600
Chr10	38589620	38592758	Glyma.10G150700
Chr10	38596951	38599004	Glyma.10G150800
Chr10	38601439	38604927	Glyma.10G150900
Chr10	38606877	38609056	Glyma.10G151000
Chr10	38607998	38608397	Glyma.10G151100
Chr10	38624314	38625496	Glyma.10G151200
Chr10	38639217	38640081	Glyma.10G151300
Chr10	38643032	38644885	Glyma.10G151400
Chr10	38650417	38660042	Glyma.10G151500
Chr10	38652436	38652753	Glyma.10G151600
Chr10	38653075	38653467	Glyma.10G151700
Chr10	38566775	38570603	Glyma.10G150300
Chr10	38580404	38584543	Glyma.10G150500
Chr11	34394104	34401757	Glyma.11G253100
Chr11	34413583	34414431	Glyma.11G253300
Chr11	34415863	34417668	Glyma.11G253400
Chr11	34428083	34434022	Glyma.11G253600
Chr11	34434822	34437768	Glyma.11G253700
Chr11	34447365	34449046	Glyma.11G253900
Chr11	34476450	34479062	Glyma.11G254200
Chr11	34480075	34483869	Glyma.11G254300
Chr11	34385927	34393054	Glyma.11G253000
Chr11	34404329	34410760	Glyma.11G253200
Chr11	34419585	34426107	Glyma.11G253500
Chr11	34438794	34440580	Glyma.11G253800
Chr11	34453500	34457695	Glyma.11G254000
Chr11	34463344	34474704	Glyma.11G254100
Chr14	48039358	48039935	Glyma.14G216000

Chr	Start	End	LOCUS TAG
Chr14	48040552	48043792	Glyma.14G216100
Chr14	48062215	48066980	Glyma.14G216200
Chr14	48088714	48091531	Glyma.14G216300
Chr14	48103216	48105558	Glyma.14G216400
Chr15	869245	874642	Glyma.15G011400
Chr15	892118	895716	Glyma.15G011500
Chr15	896377	898688	Glyma.15G011600
Chr15	903819	911563	Glyma.15G011800
Chr15	936268	944374	Glyma.15G012000
Chr15	964629	966624	Glyma.15G012100
Chr15	976439	980069	Glyma.15G012200
Chr15	991745	994732	Glyma.15G012400
Chr15	995453	998947	Glyma.15G012500
Chr15	1002510	1004331	Glyma.15G012700
Chr15	898424	902078	Glyma.15G011700
Chr15	980507	987672	Glyma.15G012300
Chr15	999019	999716	Glyma.15G012600
Chr15	1012651	1016161	Glyma.15G012800
Chr15	1031541	1035733	Glyma.15G012900
Chr15	914542	921313	Glyma.15G011900
Chr15	1036592	1049468	Glyma.15G013000
Chr15	6723421	6725900	Glyma.15G087300
Chr15	6730326	6733138	Glyma.15G087400
Chr15	6735173	6739839	Glyma.15G087500
Chr15	6741734	6746357	Glyma.15G087600
Chr15	6746643	6749503	Glyma.15G087700
Chr15	6750665	6754479	Glyma.15G087900
Chr15	6758394	6760811	Glyma.15G088000
Chr15	6774121	6782320	Glyma.15G088200
Chr15	6783646	6785019	Glyma.15G088300
Chr15	6785669	6788155	Glyma.15G088400
Chr15	6800557	6806758	Glyma.15G088500
Chr15	6835991	6836155	Glyma.15G088700
Chr15	6836966	6838087	Glyma.15G088800
Chr15	6840247	6843393	Glyma.15G088900
Chr15	6844050	6846520	Glyma.15G089000
Chr15	6853145	6857094	Glyma.15G089100
Chr15	6861152	6864731	Glyma.15G089200
Chr15	6871510	6874512	Glyma.15G089300
Chr15	6882106	6884858	Glyma.15G089400
Chr15	6885470	6889736	Glyma.15G089500
Chr15	6750138	6750578	Glyma.15G087800
Chr15	6764321	6771535	Glyma.15G088100
Chr15	6826179	6835890	Glyma.15G088600
Chr15	50912530	50915606	Glyma.15G271900
Chr15	50926598	50933550	Glyma.15G272000
Chr15	50944579	50946729	Glyma.15G272100
Chr15	50951794	50953900	Glyma.15G272200
Chr15	50965263	50965748	Glyma.15G272300
Chr15	50978413	50986557	Glyma.15G272400
Chr15	50993941	50994141	Glyma.15G272500
Chr15	51000250	51001037	Glyma.15G272600
Chr15	51008520	51009317	Glyma.15G272700
Chr15	50891554	50910117	Glyma.15G271800
Chr15	51057301	51057606	Glyma.15G273100
Chr15	51060151	51062727	Glyma.15G273200
Chr15	51069984	51071456	Glyma.15G273300
Chr15	51080478	51083012	Glyma.15G273400
Chr15	51101848	51117580	Glyma.15G273500

Chr	Start	End	LOCUS TAG
Chr15	51130117	51134021	Glyma.15G273600
Chr15	51138129	51141953	Glyma.15G273700
Chr15	51142368	51143580	Glyma.15G273800
Chr15	51181070	51183538	Glyma.15G274100
Chr15	51187965	51190100	Glyma.15G274200
Chr15	51194531	51196221	Glyma.15G274300
Chr15	51221837	51225805	Glyma.15G274400
Chr15	51235879	51244293	Glyma.15G274500
Chr15	51048375	51054499	Glyma.15G273000
Chr15	51148949	51154103	Glyma.15G273900
Chr15	51156044	51170334	Glyma.15G274000
Chr16	16915253	16919205	Glyma.16G095300
Chr16	16919328	16920316	Glyma.16G095400
Chr16	17008529	17012442	Glyma.16G095600
Chr16	16911412	16924907	Glyma.16G095200
Chr16	16973143	16981658	Glyma.16G095500
Chr16	17327427	17331403	Glyma.16G095700
Chr18	53134579	53141140	Glyma.18G243100
Chr18	53146508	53149688	Glyma.18G243200
Chr18	53151480	53157909	Glyma.18G243300
Chr18	53158882	53161134	Glyma.18G243400
Chr18	53164046	53164207	Glyma.18G243600
Chr18	53169347	53174542	Glyma.18G243700
Chr18	53176043	53177130	Glyma.18G243800
Chr18	53188685	53193206	Glyma.18G244000
Chr18	53204726	53205598	Glyma.18G244200
Chr18	53208864	53210903	Glyma.18G244300
Chr18	53223323	53229990	Glyma.18G244500
Chr18	53236145	53240803	Glyma.18G244600
Chr18	53162330	53167391	Glyma.18G243500
Chr18	53177848	53183666	Glyma.18G243900
Chr18	53193558	53202738	Glyma.18G244100
Chr18	53211659	53216296	Glyma.18G244400
Chr18	53518644	53525249	Glyma.18G248200
Chr18	53526586	53534541	Glyma.18G248300
Chr18	53546286	53547227	Glyma.18G248500
Chr18	53552136	53557152	Glyma.18G248600
Chr18	53557982	53558604	Glyma.18G248700
Chr18	53560798	53561939	Glyma.18G248800
Chr18	53562422	53564909	Glyma.18G248900
Chr18	53565060	53566525	Glyma.18G249000
Chr18	53566790	53568289	Glyma.18G249100
Chr18	53571953	53573976	Glyma.18G249200
Chr18	53578398	53580345	Glyma.18G249400
Chr18	53539192	53544229	Glyma.18G248400
Chr18	53574959	53577787	Glyma.18G249300
Chr18	53591327	53592182	Glyma.18G249500
Chr18	53603771	53609690	Glyma.18G249600
Chr19	3441407	3445044	Glyma.19G027500
Chr19	3477569	3481977	Glyma.19G027800
Chr19	3507265	3508515	Glyma.19G028000
Chr19	3511914	3519141	Glyma.19G028100
Chr19	3522717	3523287	Glyma.19G028300
Chr19	3543824	3555013	Glyma.19G028400
Chr19	3556053	3559202	Glyma.19G028500
Chr19	3567731	3570894	Glyma.19G028600
Chr19	3572652	3574919	Glyma.19G028700
Chr19	3578019	3582220	Glyma.19G028800
Chr19	3586804	3587277	Glyma.19G028900

Chr	Start	End	LOCUS TAG
Chr19	3593924	3598821	Glyma.19G029000
Chr19	3630330	3631310	Glyma.19G029200
Chr19	3647595	3650393	Glyma.19G029300
Chr19	3658795	3668005	Glyma.19G029400
Chr19	3708611	3710562	Glyma.19G029700
Chr19	3724518	3729860	Glyma.19G029800
Chr19	3729213	3729434	Glyma.19G029900
Chr19	3730272	3731974	Glyma.19G030000
Chr19	3732644	3733945	Glyma.19G030100
Chr19	3737248	3740094	Glyma.19G030300
Chr19	3752277	3756120	Glyma.19G030400
Chr19	3779017	3781453	Glyma.19G030500
Chr19	3789223	3789426	Glyma.19G030600
Chr19	3454319	3460116	Glyma.19G027600
Chr19	3471299	3475645	Glyma.19G027700
Chr19	3485283	3498153	Glyma.19G027900
Chr19	3522368	3525539	Glyma.19G028200
Chr19	3601041	3608100	Glyma.19G029100
Chr19	3681517	3690884	Glyma.19G029600
Chr19	3734989	3736738	Glyma.19G030200
Chr19	3669589	3673886	Glyma.19G029500

Suppl. Table S2 Intersection genes of GWAS candidate gene and DEGs

gene_id	regulate	nr	go	KO_id	KO_name	paths
Glyma.11G253300	down	XP_003538534.1(cysteine proteinase inhibitor B [Glycine max])	GO:0004869(molecular_function:cysteine-type endopeptidase inhibitor activity); GO:0016021(cellular_component:integral component of membrane)	K13899	CST3	
Glyma.19G028100	up	XP_006603900.1(probable galacturonosyltransferase 13 isoform X1 [Glycine max])	GO:0016021(cellular_component:integral component of membrane); GO:0000139(cellular_component:Golgi membrane); GO:0045489(biological_process:pectin biosynthetic process); GO:0071555(biological_process:cell wall organization); GO:0016757(molecular_function:transferase activity, transferring glycosyl groups); GO:0047262(molecular_function:polygalacturonate 4-alpha-galacturonosyltransferase activity)	K20867	GAUT12S	
Glyma.19G030500	up	NP_001236819.1(malonyl-CoA:isoflavone 7-O-glucoside-6'-O-malonyltransferase [Glycine max])	GO:0047164(molecular_function:isoflavone-7-O-beta-glucoside 6"-O-malonyltransferase activity); GO:0016747(molecular_function:transferase activity, transferring acyl groups other than amino-acyl groups)	K13264	IF7MAT	map00943(Isoflavonoid biosynthesis); map00944(Flavone and flavonol biosynthesis)
Glyma.15G088300	down	KRH11089.1(hypothetical protein GLYMA_15G088300, partial [Glycine max])	GO:0016021(cellular_component:integral component of membrane)			
Glyma.15G011500	down	XP_003546196.1(ADP,ATP carrier protein 1, chloroplastic [Glycine max])	GO:0031969(cellular_component:chloroplast membrane); GO:0016021(cellular_component:integral component of membrane); GO:0006810(biological_process:transport); GO:0005471(molecular_function:ATP:ADP antiporter activity); GO:0005524(molecular_function:ATP binding)	K03301	TC.AAA	
Glyma.11G254300	down	XP_003538530.1(selenium-binding protein 1 [Glycine max])	GO:0008430(molecular_function:selenium binding); GO:0004721(molecular_function:phosphoprotein phosphatase activity)	K17285	SELENBP1	map00920(Sulfur metabolism)
